# The Influence of Increased Prepregnancy Body Mass Index and Excessive Gestational Weight Gain on Pregnancy Course and Fetal and Maternal Perinatal Outcomes

**DOI:** 10.3390/healthcare8040362

**Published:** 2020-09-24

**Authors:** Milan Lackovic, Dejan Filimonovic, Sladjana Mihajlovic, Biljana Milicic, Ivana Filipovic, Marija Rovcanin, Dejan Dimitrijevic, Dejan Nikolic

**Affiliations:** 1 Clinical Hospital Center “Dr. Dragiša Mišović”, 11000 Belgrade, Serbia; milan.lackovic@dragisamisovic.bg.ac.rs (M.L.); mihajlovicobg@gmail.com (S.M.); ivana.filipovic@dragisamisovic.bg.ac.rs (I.F.); 2 Faculty of Medicine, University of Belgrade, 11000 Belgrade, Serbia; ginekologija.misovic@gmail.com (D.F.); rovcanin.marija@gakfront.org (M.R.); dimitrijevic.dejan@gakfront.org (D.D.); 3 Obstetrics/Gynecology Clinic “Narodni front”, 11000 Belgrade, Serbia; 4 Faculty of Dental Medicine, University of Belgrade, 11000 Belgrade, Serbia; biljana.milicic@sbb.rs; 5 Physical Medicine and Rehabilitation Department, University Children’s Hospital, Tirsova 10, 11000 Belgrade, Serbia

**Keywords:** obesity, weight gain, pregnancy, perinatal outcome, early motor development

## Abstract

*Background*: The aim of our study was to assess the influence of prepregnancy Body Mass Index (BMI) changes as well as excessive gestational weight gain (GWG) on maternal and fetal perinatal parameters. Furthermore, we aimed to analyze the influence of increased prepregnancy BMI values and excessive GWG on neonatal early motoric development. *Methods*: The 203 eligible female participants were evaluated. Prepregnancy Body Mass Index (BMI) and excessive gestational weight gain (GWG) defined according to Institute of Medicine 2009 guidelines in the USA were assessed with tested maternal and fetal perinatal parameters and infants early motoric development (Alberta Infant Motor Scale—AIMS). *Results*: Significant predictors of increased prepregnancy BMI in perinatal period include: weight at delivery (*p* = 0.001), GWG (*p* = 0.002) and BMI at delivery (*p* < 0.001), while significant predictors of excessive GWG in perinatal period are: prepregnancy BMI (*p* = 0.029) and BMI at delivery (*p* < 0.001). In the group of participants with both increased prepregnancy BMI and excessive GWG versus others, significant predictors were: hypertension (HTA) (*p* = 0.019), amniotic fluid index (AFI) (*p* = 0.047), Pronation (AIMS) (*p* = 0.028) and Supination (AIMS) (*p* = 0.029). *Conclusion*: Increased prepregnancy BMI and excessive GWG are significantly associated with numerous perinatal factors that could alter the pregnancy course, pregnancy outcome and early motoric development of newborn. Moreover, increased prepregnancy BMI is shown to be a significant predictor of excessive GWG; thus, early selection of pregnant women for close monitoring of weight gain during pregnancy will have positive effects on reducing the risk of less favorable pregnancy course and early motoric development of newborn.

## 1. Introduction

Obesity and overweight are significant public health issues. According to the World Health Organization (WHO) in 2016, more than 1.9 billion adults aged 18 years and older were overweight. The worldwide prevalence of obesity nearly tripled between 1975 and 2016 [1]. The number of obese and overweight mothers is also increasing, especially in urban areas. Several definitions concerning maternal obesity have been proposed including Body Mass Index (BMI) categories, increased waist circumference and waist-to-hip ratio as well as absolute measures such as gravid weight above 200lb, but final consensus has not been adopted yet [2,3].

Maternal obesity and perinatal outcomes are also determined with gestational weight gain (GWG). Excessive GWG potentially increases perinatal complications such as Cesarean delivery, large-for-gestational-age, macrosomia and childhood overweight or obesity for the offspring. This is defined according to the Institute of Medicine 2009 gestational weight gain guideline in the USA [4]. Increased prepregnancy BMI and excessive (GWG) are potentially modifiable risk factors. Prevention of or reduction in these factors may decrease unfavorable perinatal and neonatal outcomes, supporting the Development Origin of Health and Disease (DOHaD) hypothesis. This hypothesis emphases an unfavorable in utero environment on fetal and neonatal development, pointing to the greater risk of the disease development in the adulthood period [5].

Obesity is considered as a systemic inflammatory condition that might affect insulin resistance, type II diabetes and hypertension among other conditions [6].

Increased prepregnancy BMI and excessive GWG increase the risk of numerous pregnancy complications [7,8,9,10]. Increased maternal BMI is considered as an important predictor of maternal and newborn health, including but not limited to the increased risk of poor neurodevelopmental newborn disorders, leading to various degrees of cognitive disability, developmental delay, impaired language skills as well as the cerebral palsy [11,12,13]. Moreover, higher weight gain during pregnancy predicts a neonate that is large for the gestational age, which could be marker of neonatal morbidity. However previous studies suggested that GWG is not associated with child Peabody score and Raven score [4,14].

Previous reports stated that various prenatal maternal factors including but not limited to gestational or pregestational diabetes, and overweight and obesity, might prolong child motor milestone achievements and cause neurodevelopmental delay [15,16]. The importance of early recognition of these factors along with preconceptual interventions could be effective in reducing the less favorable outcomes in the early motoric development of children.

We hypothesized that changes in prepregnancy BMI and excessive GWG are potential risk factors associated with less favorable pregnancy and neonatal outcome. Therefore, the aim of our study was to assess the influence of increased prepregnancy BMI changes as well as excessive GWG on maternal and fetal perinatal parameters. Furthermore, we aimed to analyze the influence of increased prepregnancy BMI and excessive GWG on neonatal early motoric development.

## 2. Methods

### 2.1. Study Group

**Figure 1:**
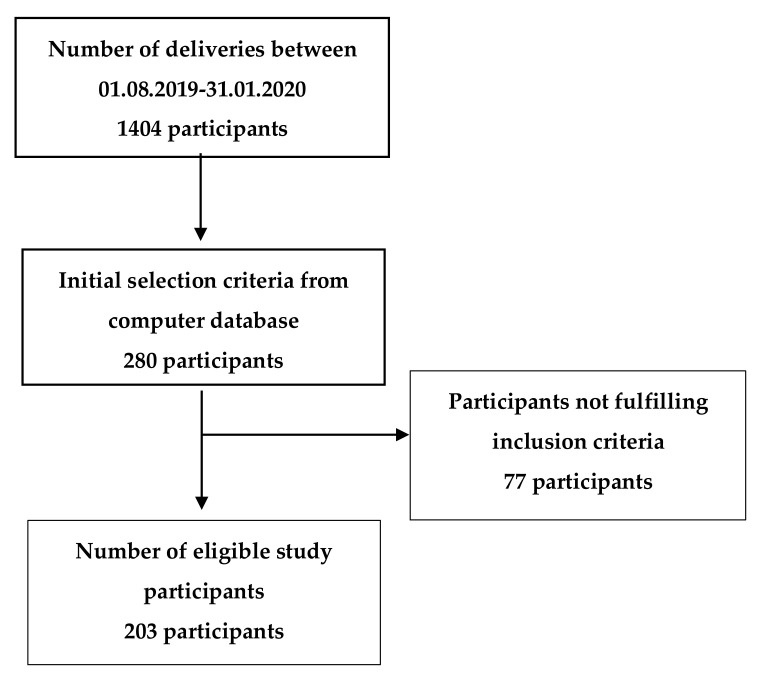
Flow chart of study participants selection.

The prospective study included 203 eligible female participants that were admitted to the Obstetrics ward for delivery between 1 August 2019 and 31 January 2020. Eligible female participants were defined as singleton pregnancies that were not conceived using assisted reproductive techniques (ART). All pregnant women in this study had low risk of chromosomal disorders based on noninvasive prenatal testing. The selection of study participants was described in a flow chart (Figure 1). To minimize the possibility of bias in the study, we established inclusion and exclusion criteria, since for pregnant women who underwent ART, several parameters might influence the possibility of the bias. According to Serbian National Regulation defining artificial reproductive technique (ART) procedures, increased BMI is considered as an exclusion criteria for ART procedures [17]. More frequent pregnancy related complications associated with frequency of advanced maternal age, multifetal pregnancies, Cesarean delivery as a more frequent delivery mode are well known complications associated with ART procedures. Due to increased adverse pregnancy and perinatal outcomes associated with ART procedures in comparison with naturally conceived pregnancies, those women were excluded from the study [18,19,20,21,22,23].

Prior inclusion in the study, participants were informed about the study protocol, and consent was obtained. The study followed the principles of good clinical practice and was approved by Institutional Review Board (01-14706/19).

The study group was randomly selected from the computer data base at the Obstetric ward of the Hospital where delivery was performed. Every fifth admitted women to the Hospital for delivery was selected.

Regarding the sample size for this study, we estimated that the hypertension (HTA) was significantly more frequent in pregnant women with excessive GWG and increased BMI than in pregnant women without excessive GWG (70.6% vs. 29.4%). Additionally, analysis of diabetes mellitus (DM), showed that DM was more prevalent in pregnant women with excessive GWG and increased BMI than in the group of pregnant women without excessive GWG (64.0% vs. 36.0%). Enrolment of 200 pregnant women (where frequency of pregnant women with excessive GWG is 34%) will achieve 97.2% power to detect a significant difference in the development of DM. Additionally, this simple size will achieve 99.3% power to detect a significant difference in HTA.

The study sample might be considered as a representative, since the maternity ward where the samples were collected is among the five most frequent in the country dealing with all types of pathology of pregnancy, where patients come from all parts of Serbia; so, in that sense, the sample is assumed to be homogeneous.

To reduce the possibility of nonrespondent bias for the design of the study, our survey was accessible, well explained and understandable, while for the study implementation, eligible participants were told that collecting data and samples for testing were mandatory, and a satisfactory response rate was achieved.

### 2.2. Evaluated Parameters

The values of prepregnancy height, weight and BMI, positive family history and presence of allergies and thrombophilia were collected from health data reports from primary health centers (women that were checked up to 3 months prior to pregnancy) where participants were regularly screened and followed-up.

Based on the WHO ranges, pregnant women were allocated in four different groups depending on their pregestational BMI: underweight, normal weight, overweight and obese. Underweight was defined as BMI bellow 18.5 kg/m^2^, normal weight as BMI between 18.5–24.9 kg/m^2^, overweight (preobesity) as BMI between 25.0–29.9 kg/m^2^ and obesity as BMI 30.0 kg/m^2^ and above [24]. Ultrasound (US) was used to evaluate fetal biparietal diameter (BPD), head circumference (HC), abdominal circumference (AC), femoral length (FL), estimated fetal weight (EFW) and amniotic fluid index (AFI) on admission to the hospital up to three days prior to delivery. All US measurements were performed by the same sonographer on the same model of US (The Voluson™ E8 ultrasound system (GE Healthcare Dharmacon, Inc., Chicago, IL, USA)). Based on BPD, AC, HC and FL, EFW was calculated using a US software system. The premature rupture of membranes (PROM) was defined as a rupture of the amniotic sac after 37 gestational weeks before the beginning of the delivery [25]. Pregnancy related hypertension (HTA) was defined as blood pressure above 140/90 mmHg, gestational diabetes mellitus (DM) as pathological values of oral glucoses tolerance test or postprandial glycaemia values. Gestational week was calculated by Naegele’s rule and transferred into days for adequate calculation in this study. The GWG is mathematically expressed as maternal prepregnancy and at delivery weight difference. During pregnancy, averagely 3.5 kg of weight gain counts as fetal weight, 0.5 kg as placenta, amniotic fluid is up to 1 kg and remaining weight gain counts as adipose tissue accumulation and extravascular fluid [26]. Excessive GWG was defined according to further parameters: depending on prepregnancy BMI values, recommended total weight gain for singleton pregnancies is 12.5–18.0 kg for underweight (BMI < 18.5 kg/m^2^), 11.5–16.0 kg for normal weight (BMI 18.5–24.9 kg/m^2^), 7.0–11.5 kg for overweight (BMI 25.0–29.9 kg/m^2^) and 5.0–9.0 kg for obese (BMI ≥ 30 kg/m^2^) [4]. All values above these ranges for weight category were considered as excessive GWG.

The blood was drawn twice: before delivery (up to 24 h before labor) and after delivery (24 h after labor). Leucocytes, hemoglobin (HGB), thrombocytes, glucose, D dimer and blood type (BT) (rhesus negative versus positive) were assessed. During the labor, complications at delivery and delivery mode (vaginal, Cesarean Section (SC), instrumental, induced) were assessed. At the beginning of delivery, three types of delivery mode were defined: spontaneous, induced and planned Cesarean Section. At the end of delivery, five types of delivery modes were defined: spontaneous, induced, assisted (instrumental), planned and emergency Cesarean Section.

Additionally, drug use in pregnancy and at delivery, positive urine culture (UC), presence of group B streptococcus (GBS) and APGAR score in the first and fifth minute were evaluated.

Early motoric development of infants was assessed by Alberta infant motor scale (AIMS) at the age of 3 months. Pronation and supination points were calculated separately. The AIMS is a nonreferenced measure with high sensitivity and specificity for detection of motor deficits. It is composed of 58 items, where pronation has 21, supination 9, sitting 12 and standing 16. Since tested newborns were aged 3 months, only pronation and supination were analyzed [27].

### 2.3. Statistical Analysis

Statistical Package for Social Science (SPSS software package, version 26.0; SPSS Inc., Chicago, IL, USA) was used for all the data analyses. Descriptive data for all groups and variables were expressed as the mean value (MV) and standard deviation (SD) (MV ± SD) for continuous measures, or absolute number (N) and percent (%) of a group for discrete measures. Comparation categorical data between groups with and without GWG were analyzed using the Pearson chi-square test. A normal distribution of all numeric data was tested using the Koglomorov–Smirnov test. If the data were normally distributed, the t-test was used to compare data between observed groups. Nonparametric data were analyzed using the Mann–Whitney U test. Multiple linear regression analysis was performed to measure the influence of all observed factors as independent to prepregnancy BMI as a dependent variable.

The association of observed parameters with GWG was analyzed using univariate and multivariable binary logistic regression methods. Differences were considered significant when *p* value was <0.05.

## 3. Results

In Table 1, tested parameter characteristics were presented. Seven (3.4%) deliveries at the beginning of delivery were induced, but ended as emergency Cesarean Section because of unsuccessful induction of labors, prolonged delivery or signs of fetal distress. A total of 11 (5.4%) deliveries started as spontaneous and ended as emergency Cesarean Sections.

In pregnant women with excessive GWG during pregnancy versus those without excessive GWG, prepregnancy BMI (*p* < 0.001), weight at delivery (*p* < 0.001), BMI at delivery (*p* < 0.001), BPD fetal (*p* = 0.026), AC fetal (*p* = 0.002), EFW (*p* = 0.004) and glucose after delivery (*p* = 0.025) were significantly higher, while Apgar in 1 min (*p* = 0.013) and in 5 min (*p* = 0.007), HGB after delivery (*p* < 0.001) as well as pronation score (*p* < 0.001) and supination score (*p* < 0.001) of AIMS were significantly lower (Table 2). Those with excessive GWG had significantly frequent positive family history (*p* = 0.024), present HTA (*p* = 0.001), DM (*p* < 0.001) anemia (*p* = 0.001), drug use in pregnancy (*p* = 0.003) and complications at delivery (*p* = 0.045).

Increased prepregnancy BMI was significantly positively associated with: weight at delivery, GWG and BMI at delivery, HTA, DM (*p* < 0.001), positive family anamnesis (*p* = 0.006), anemia (*p* = 0.030), drug use in pregnancy (*p* = 0.015), AC fetal (*p* = 0.015), EFW (*p* = 0.016), AFI (*p* = 0.019) and glucose after delivery (*p* = 0.026). The significant negative association of prepregnancy BMI was with menarche (*p* = 0.023), pronation score (*p* = 0.002) and supination score (*p* < 0.001) of AIMS (Table 3).

The excessive GWG was significantly associated with: prepregnancy BMI, BMI at delivery, obesity degree, DM, AIMS pronation and supination scores (*p* < 0.001), positive family anamnesis (*p* = 0.026), HTA (*p* = 0.004), anemia (*p* = 0.001), drug use in pregnancy (*p* = 0.003), complications at delivery (*p* = 0.050), BPD fetal (*p* = 0.043), AC fetal (*p* = 0.017), EFW (*p* = 0.010), Apgar score in 1 min (*p* = 0.047) and 5 min (*p* = 0.016), HGB before delivery (*p* = 0.019), HGB after delivery (*p* = 0.006) and glucose after delivery (*p* = 0.009) (Table 3).

The presence of both increased prepregnancy BMI and excessive GWG were significantly associated with: family anamnesis (*p* = 0.010), HTA (*p* = 0.002), DM (*p* < 0.001), Thrombophilia (*p* = 0.039), Anemia (*p* = 0.004), drug use in pregnancy (*p* = 0.010), AC fetal (*p* = 0.035), AFI (*p* = 0.002), HGB after delivery (*p* = 0.013), glucose after delivery (*p* = 0.010), D dimer after delivery (*p* = 0.039), Pronation (AIMS) (*p* < 0.001) and Supination (AIMS) (*p* < 0.001) (Table 3).

Significant predictors of increased prepregnancy BMI in perinatal period include: weight at delivery (*p* = 0.001), GWG (*p* = 0.002) and BMI at delivery (*p* < 0.001), while significant predictors of excessive GWG in perinatal period are: prepregnancy BMI (*p* = 0.029) and BMI at delivery (*p* < 0.001). In the group of participants with both increased prepregnancy BMI and excessive GWG versus others, significant predictors were: HTA (*p* = 0.019), AFI (*p* = 0.047), Pronation (AIMS) (*p* = 0.028) and Supination (AIMS) (*p* = 0.029) (Table 4).

## 4. Discussion

In our study, we have shown that increased prepregnancy BMI or excessive GWG were significantly associated with BMI at delivery, HTA, DM, positive family history, anemia, drug use in pregnancy, ultrasound parameters (AC and EFW) as well as pronation and supination score of AIMS. Furthermore, only increased prepregnancy BMI was significantly associated with weight at delivery, AFI, glucose levels after delivery and menarche. This is to the certain degree in line with previous reports, where women with increased prepregnancy BMI tend to gain weight [28,29]. In the study of Dong et al., elevated prepregnancy BMI and excessive GWG in first trimester of pregnancy were associated with gestational DM [30]; however, Yong et al., reported that GWG nor in the first neither in the second trimester of pregnancy was independently associated with the risk for gestational DM [31]. In our study, only excessive GWG was significantly associated with obesity degree, complications at delivery, ultrasound BPD parameter, Apgar scores in 1 and 5 min, HGB levels bout before and after delivery and glucose levels after delivery. Moreover, both increased prepregnancy BMI and excessive GWG were significantly associated with family history, HTA, DM, thrombophilia, anemia, drug use in pregnancy, AC fetal, AFI, HGB, glucose and D dimer after delivery as well as pronation and supination score of AIMS.

These findings clearly point to the fact that both increased prepregnancy BMI and/or excessive GWG are associated with numerous factors that can influence the course of pregnancy as well as early infant motoric development. However both parameters should be assessed individually along with their synergistic effects, since some of the tested perinatal parameters are found to be influenced by one or another variable.

In previous reports, both obesity and GWG are shown to have associations with pregnancy outcomes [32,33,34,35], bearing in mind that their mechanisms are still unclear particularly for GWG [32]. Additionally, it should be pointed that both obesity and GWG might have individual as well as cumulative effects not only on various pregnancy outcome factors but on their severity degree as well [36,37,38]. Our findings have shown that participants with increased prepregnancy BMI had 14 times more chance of weight gain at delivery, over one and a half times more chance of being anemic and over three and a half times more chance of increased glucose levels. Furthermore, participants with both increased prepregnancy BMI and excessive GWG have over seven times more chance of having HTA, as well as over two and a half times more chance of lower scores for pronation and supination of AIMS. Thus, the importance of weight gain control in pregnant woman that are obese could positively alter pregnancy outcomes [38].

Furthermore, we have shown multidimensional effects of both increased prepregnancy BMI and excessive GWG on the tested perinatal parameters as well as early motoric development. Previous reports demonstrated unfavorable obesity effects on pregnancy, pregnancy outcome as well as labor [32,39,40]. This might imply the possible assumption that various physiological and regulatory mechanisms might be affected by these two tested variables and in different capacities, thus affecting not only pregnancy course but its outcome.

Furthermore, our findings point to the fact that tested obese women are more prone to have an increased chance of excessive GWG, thus emphasizing the need for regular checkups during the entire course of pregnancy not only for early detection of possible complications but also to treat and prevent progression. In the study of Groth et al., potential genetic association with high GWG was noticed in obese black women [41].

There are several limitations to this study. The tested participants belong to the Serbian population. Given the fact that specific socio-economic variations and genetic dispositions could exist among different populations, further studies are needed. In this study, for the estimation of the obesity degree, waist circumference (WC) and waist to hip ratio (WHR) of pregnant women were not analyzed. Additionally, nonalcoholic fatty liver disease (NAFLD) that might be associated with obesity [42] along with liver function parameters should be evaluated. Another limiting factor refers to the number of participants; thus, additional studies on larger samples are advised. The need for a larger number of patients would lead to the better understanding and detection of interindividual variability of individual parameters, or detection of variability in a larger proportion of eligible participants. Furthermore, the accuracy of ultrasound scan should be taken into consideration.

## 5. Conclusions

Increased prepregnancy BMI and excessive GWG are significantly associated with numerous perinatal risk factors that could alter the pregnancy course, pregnancy outcome and early motoric development of newborn. Moreover, increased prepregnancy BMI is shown to be significant predictor of excessive GWG; thus, early selection of pregnant women for close monitoring of weight gain during pregnancy will have positive effects on reducing the risk of less favorable pregnancy course and early motoric development of newborn.

Finally, these findings will additionally contribute to the better understanding of the potential role of increased prepregnancy BMI and excessive GWG on perinatal risk factors and early motoric development of newborn, adding additional knowledge to practitioners in clinical and primary health care settings for establishing appropriate clinical decision-making strategies. Therefore, maternal obesity could be a significant public health burden; thus, preventive and educational programs along with continuous monitoring during pregnancy will have a positive impact on adverse maternal and fetal outcomes.

Additionally, the strength of the study refers to the fact that a wide range of parameters were analyzed and the influence of pregnant women BMI and GWG on them. Moreover, the study sample was drawn from general population including all socioeconomic statuses.

## Figures and Tables

**Table 1:** Tested parameters characteristics (*N* = 203).

Variables		Value
Age, (years) (MV ± SD)		31.98 ± 4.82
Height, (cm) (MV ± SD)		169.97 ± 5.77
Prepregnancy BMI, (kg/m^2^) (MV ± SD)		23.32 ± 3.74
Preconceptional weight, (kg) (MV ± SD)		67.47 ± 11.49
BMI (kg/m^2^) at delivery (MV ± SD)		28.86 ± 4.35
Weight at delivery, (kg) (MV ± SD)		83.40 ± 13.01
Menarche, (years) (MV ± SD)		13.04 ± 1.21
Gestational week, (days) (MV ± SD)		275.51 ± 8.62
BDP fetal (mm) (MV ± SD)		94.74 ± 3.62
HC fetal (mm) (MV ± SD)		338.09 ± 26.11
AC fetal (mm) (MV ± SD)		344.53 ± 21.0
FL fetal (mm) (MV ± SD)		74.26 ± 4.07
EFW (grams) (MV ± SD)		3494.06 ± 453.06
AFI (mm) (MV ± SD)		127.41 ± 37.97
Newborn length (cm) (MV ± SD)		52.85 ± 2.36
Newborn weight (grams) (MV ± SD)		3508.03 ± 502.43
APGAR 1 min (MV ± SD)		8.67 ± 0.71
APGAR 5 min (MV ± SD)		9.75 ± 0.51
Leucocytes (10^9^/L) before delivery (MV ± SD)		9.92 ± 2.28
HGB (gr/L) before delivery (MV ± SD)		119.15 ± 9.67
Thrombocytes (10^9^/L) before delivery (MV ± SD)		227.99 ± 59.20
Glucose (mmol/L) before delivery (MV ± SD)		4.62 ± 0.68
D dimer (mg/L) before delivery (MV ± SD)		1.70 ± 0.99
Leucocytes (10^9^/L) after delivery (MV ± SD)		10.96 ± 2.72
HGB (gr/L) after delivery (MV ± SD)		105.09 ± 11.74
Thrombocytes (10^9^/L) after delivery (MV ± SD)		211.75 ± 58.58
Glucose (mmol/L) after delivery (MV ± SD)		5.07 ± 0.94
D dimer (mg/L) after delivery (MV ± SD)		1.46 ± 0.81
Pronation (AIMS) (MV ± SD)		2.61 ± 0.56
Supination (AIMS) (MV ± SD)		2.71 ± 0.49
Excessive weight gain (kg), N (%)		92 (45.3)
Parity, *N* (%)	1	111 (54.7)
	2	79 (38.9)
	3	11 (5.4)
	4	2 (1.0)
Obesity degree by BMI (kg/m^2^), *N* (%)	Underweight	5 (2.5)
	Normal weight	114 (56.2)
	Overweight	71 (35.0)
	Obese	13 (6.4)
Allergies, *N* (%)		22 (10.8)
Family history, *N* (%)		41 (20.2)
Hypertension, *N* (%)		17 (8.4)
Diabetes mellitus, *N* (%)		50 (24.6)
Thrombophilia, *N* (%)		13 (6.4)
Anemia, *N* (%)		89 (43.8)
Drug use in pregnancy, *N* (%)		125 (61.6)
BT (Rhesus negative), *N* (%)		27 (13.3)
UC, *N* (%)	Positive	21 (10.3)
GBS, *N* (%)		35 (17.2)
Drug use at delivery, *N* (%)		62 (30.5)
Delivery mode, *N* (%)	Vaginal	128 (63.1)
	Cesarean section	55 (27.1)
	Instrumental	13 (6.4)
	Induced	7 (3.4)
PROM, *N* (%)		24 (11.9)
Complications at delivery, *N* (%)		25 (12.3)
IUGR, *N* (%)		14 (6.9)

BMI—body mass index; BDP—fetal biparietal diameter; HC—head circumference; AC—abdominal circumference; FL—femoral length; EFW—estimated fetal weight; AFI—amniotic fluid index; HGB—hemoglobin; AIMS—Alberta Infant Motor Scale; BT—blood type; UC—urine culture; GBS—group B streptococcus; PROM—premature rupture of membranes; IUGR—intrauterine growth restriction.

**Table 2:** Parameters characteristics due to the presence of weight gain (*N* = 203).

Variables		Excessive GWG		*p*
		Yes	No	
Age (years), (MV ± SD)		32.57 ± 4.77	31.50 ± 4.82	0.115 **
Prepregnancy BMI (kg/m^2^)		25.86 ± 3.44	21.21 ± 2.45	<0.001 ***
Preconceptional weight (kg), (MV ± SD)		93.73 ± 9.99	74.85 ± 8.08	<0.001 ***
Obesity degree by BMI (kg/m^2^), *N* (%)	Underweight	1 (20)		<0.001 ****
	Normal weight	19 (16.7)		
	Overweight	61 (85.9)		
	Obesity	11 (84.6)		
BMI (kg/m^2^) at delivery		32.26 ± 3.58	26.05 ± 2.53	<0.001 ***
Parity, *N* (%)	1	55 (49.5)		0.591 ****
	2	32 (40.5)		
	3	4 (36.4)		
	4	1 (50.0)		
Allergies ^1^, *N* (%)		11 (50)	11 (50)	0.641 ****
Family history ^2^, *N* (%)		25 (61)	16 (39)	0.024 ****
HTA ^1^, *N* (%)		14 (82.4)	3 (17.6)	0.001 ****
DM ^1^, *N* (%)		36 (72.0)	14 (28.0)	<0.001 ****
Thrombophilia ^1^, *N* (%)		8 (61.5)	5 (38.5)	0.225 ****
Anemia ^1^, *N* (%)		52 (58.4)	37 (41.6)	0.001 ****
Drug use in pregnancy ^1^, *N* (%)		67 (53.6)	58 (46.4)	0.003 ****
Menarche, (years), (MV ± SD)		12.97 ± 0.94	13.11 ± 1.38	0.711 ***
BT ^1^, *N* (%)		11 (40.7)	16 (59.3)	0.608 ****
UC ^2^, *N* (%)		8 (38.1)	13 (61.9)	0.482 ****
GBS ^2^, *N* (%)		12 (34.3)	23 (65.7)	0.149 ****
Drug use at delivery, *N* (%)		34 (54.8)	28 (45.2)	0.071 ****
Delivery mode, *N* (%)	Vaginal	51 (39.8)		0.239 ****
	Cesarean section	30 (54.5)		
	Instrumental	7 (53.8)		
	Induced	4 (57.1)		
PROM ^1^, *N* (%)		15 (62.5)	9 (37.5)	0.067 ****
Complications at delivery ^1^, *N* (%)		16 (64)	9 (36)	0.045 ****
Gestational week, (MV ± SD)		275.17 ± 9.32	275.79 ± 8.02	0.820 ***
BPD fetal (mm), (MV ± SD)		95.32 ± 3.77	94.27 ± 3.43	0.026 ***
HC fetal (mm), (MV ± SD)		337.68 ± 14.95	338.42 ± 32.67	0.407 ***
AC fetal (mm), (MV ± SD)		348.48 ± 22.60	341.25 ± 19.24	0.002 ***
FL fetal (mm), (MV ± SD)		74.64 ± 3.48	73.95 ± 4.49	0.144 ***
EFW (grams), (MV ± SD)		3585.38 ± 481.19	3418.38 ± 415.49	0.004 ***
AFI (mm), (MV ± SD)		132.93 ± 44.70	122.84 ± 30.78	0.096 ***
IUGR ^1^, *N* (%)		5 (35.7)	9 (64.3)	0.454 ****
Newborn length (cm), (MV ± SD)		53.09 ± 2.56	52.65 ± 2.17	0.208 ***
Newborn weight (grams), (MV ± SD)		3576.41 ± 542.90	3451.35 ± 461.06	0.077 *
Apgar 1 min, (MV ± SD)		8.55 ± 0.78	8.76 ± 0.64	0.013 ***
Apgar 5 min, (MV ± SD)		9.65 ± 0.56	9.83 ± 0.45	0.007 ***
Leucocytes (10^9^ g/L) before delivery, (MV ± SD)		10.21 ± 2.49	9.68 ± 2.06	0.069 ***
HGB (gr/L) before delivery, (MV ± SD)		117.38 ± 10.11	120.64 ± 9.07	0.017 *
Thrombocytes (10^9^ g/L) before delivery, (MV ± SD)		232.84 ± 60.47	223.89 ± 58.06	0.363 ***
Glucose (mmol/L) before delivery, (MV ± SD)		4.67 ± 0.67	4.58 ± 0.68	0.233 ***
D dimer (mg/L) before delivery, (MV ± SD)		1.70 ± 0.90	1.71 ± 1.06	0.859 ***
Leucocytes (10^9^ g/L) after delivery, (MV ± SD)		11.20 ± 2.75	10.75 ± 2.69	0.158 ***
HGB (gr/L) after delivery, (MV ± SD)		102.57 ± 11.14	107.23 ± 11.86	<0.001 ***
Thrombocytes (10^9^ g/L) after delivery, (MV ± SD)		212.71 ± 60.80	210.94 ± 56.91	0.935 ***
Glucose (mmol/L) after delivery, (MV ± SD)		5.26 ± 1.06	4.91 ± 0.79	0.025 ***
D dimer (mg/L) after delivery, (MV ± SD)		1.51 ± 0.85	1.42 ± 0.77	0.529 ***
Pronation (AIMS), (MV ± SD)		2.40 ± 0.65	2.75 ± 0.46	<0.001 ***
Supination (AIMS), (MV ± SD)		2.49 ± 0.58	2.86 ± 0.35	<0.001 ***

GWG—gestational weight gain; BMI—body mass index; HTA—hypertension; DM—diabetes mellitus; BT—blood type; UC—urine culture; GBS—group B streptococcus; PROM—premature rupture of membranes; BDP—fetal biparietal diameter; HC—head circumference; AC—abdominal circumference; FL—femoral length; EFW—estimated fetal weight; AFI—amniotic fluid index; IUGR—intrauterine growth restriction; HGB—hemoglobin; AIMS—Alberta Infant Motor Scale; ** Independent Samples *t* Test; *** Mann–Whitney U Test; **** Chi-Square Test; ^1^ Present; ^2^ Positive.

**Table 3:** Regression analysis of perinatal parameters for increased prepregnancy BMI and excessive GWG.

Parameters	Linear Regression Analysis Increased Prepregnancy BMI			Logistic Regression Analysis Excessive GWG			Logistic Regression Analysis Increased Prepregnancy BMI and Excessive GWG		
	B	95% CI	*p*	Exp(B)	95% CI	*p*	Exp(B)	95% CI	*p*
Age (years)	0.112	0.006–0.219	0.039	1.048	0.988–1.111	0.116	1.042	0.980–1.108	0.193
Weight at delivery (kg)	0.250	0.231–0.270	<0.001	-	-	-	-	-	-
Prepregnancy BMI (kg/m^2^)	-	-	-	1.607	1.419–1.821	<0.001	-	-	-
GWG (kg)	4.653	3.836–5.471	<0.001	-	-	-	-	-	-
BMI (kg/m^2^) at delivery	0.793	0.746–0.840	<0.001	1.802	1.551–2.092	<0.001	-	-	-
Parity	−0.584	−1.384–0.216	0.152	0.766	0.494–1.186	0.232	0.702	0.436–1.131	0.146
Obesity degree	-	-	-	15.721	7.769–31.811	<0.001	-	-	-
Allergies	0.860	−0.806–2.525	0.310	1.235	0.509–2.993	0.641	1.124	0.447–2.826	0.804
Family anamnesis	1.775	0.506–3.045	0.006	2.215	1.099–4.466	0.026	2.494	1.239–5.017	0.010
HTA	5.179	3.449–6.909	<0.001	6.462	1.796–23.252	0.004	5.432	1.828–16.136	0.002
DM	2.641	1.493–3.788	<0.001	4.454	2.213–8.965	<0.001	5.574	2.807–11.066	<0.001
Thrombophilia	2.025	−0.076–4.127	0.059	2.019	0.637–6.399	0.233	3.384	1.063–10.773	0.039
Anemia	1.143	0.109–2.177	0.030	2.600	1.469–4.601	0.001	2.393	1.322–4.331	0.004
Drug use in pregnancy	1.304	0.252–2.356	0.015	2.449	1.356–4.424	0.003	2.297	1.216–4.340	0.010
Menarche	−0.496	−0.924–−0.069	0.023	0.906	0.716–1.146	0.409	0.851	0.658–1.100	0.218
BT	−1.159	−2.679–0.361	0.134	0.806	0.354–1.836	0.608	0.644	0.258–1.606	0.345
UC	0.929	−0.771–2.628	0.283	0.718	0.284–1.815	0.484	1.221	0.480–3.103	0.675
GBS	−0.763	−2.133–0.608	0.274	0.574	0.268–1.228	0.153	0.623	0.274–1.416	0.259
Drug use at delivery	0.039	−1.088–1.166	0.946	1.738	0.952–3.173	0.072	1.490	0.801–2.769	0.208
Delivery mode	0.589	−0.084–1.263	0.086	1.398	0.966–2.022	0.075	1.352	0.934–1.957	0.111
PROM	0.860	−0.746–2.467	0.292	2.237	0.930–5.383	0.072	2.179	0.921–5.150	0.076
Complications at delivery	0.320	−1.259–1.899	0.690	2.386	1.001–5.689	0.050	1.626	0.695–3.802	0.262
Gestational week at delivery	−0.019	−0.080–0.041	0.529	0.992	0.960–1.024	0.610	0.982	0.950–1.015	0.285
BPD fetal (mm)	0.116	−0.027–0.259	0.111	1.088	1.003–1.180	0.043	1.017	0.937–1.103	0.691
HC fetal (mm)	0.000	−0.020–0.020	0.992	0.999	0.988–1.010	0.841	0.998	0.986–1.010	0.731
AC fetal (mm)	0.030	0.006–0.055	0.015	1.018	1.003–1.032	0.017	1.016	1.001–1.032	0.035
FL fetal (mm)	0.089	−0.038–0.217	0.168	1.048	0.970–1.133	0.237	1.063	0.972–1.162	0.182
EFW (grams)	0.001	0.000–0.003	0.016	1.001	1.000–1.002	0.010	1.001	1.000–1.001	0.090
AFI (mm)	0.016	0.003–0.030	0.019	1.007	1.000–1.015	0.065	1.014	1.005–1.023	0.002
IUGR	−0.021	−2.070–2.027	0.984	0.651	0.210–2.016	0.457	0.763	0.230–2.528	0.658
Newborn length (cm)	0.100	−0.120–0.321	0.370	1.084	0.961–1.222	0.189	1.065	0.939–1.208	0.329
Newborn weight (grams)	0.001	0.000–0.002	0.255	1.001	1.000–1.001	0.080	1.000	1.000–1.001	0.313
APGAR 1 min	−0.523	−1.255–0.208	0.160	0.661	0.439–0.995	0.047	0.813	0.546–1.211	0.308
APGAR 5 min	−0.872	−1.888–0.145	0.092	0.494	0.277–0.878	0.016	0.630	0.362–1.097	0.102
Leucocytes (10^9^/L) before delivery	0.074	−0.155–0.302	0.526	1.111	0.980–1.258	0.100	1.106	0.974–1.256	0.119
HGB (gr/L) before delivery	−0.042	−0.096–0.011	0.121	0.965	0.936–0.994	0.019	0.972	0.942–1.002	0.064
Thrombocytes (10^9^/L) before delivery	0.006	−0.003–0.014	0.206	1.003	0.998–1.007	0.286	1.005	1.000–1.010	0.068
Glucose (mmol/L) before delivery	0.144	−0.628–0.916	0.713	1.229	0.812–1.858	0.329	1.233	0.804–1.891	0.337
D dimer (mg/L) before delivery	0.234	−0.294–0.762	0.384	0.988	0.745–1.310	0.932	1.155	0.866–1.541	0.327
Leucocytes (10^9^/L) after delivery	0.046	−0.145–0.238	0.634	1.062	0.959–1.177	0.249	1.044	0.939–1.161	0.426
HGB (gr/L) after delivery	−0.057	−0.100–−0.013	0.012	0.965	0.941–0.990	0.006	0.968	0.944–0.993	0.013
Thrombocytes (10^9^/L) after delivery	0.002	−0.007–0.011	0.712	1.001	0.996–1.005	0.831	1.001	0.997–1.006	0.555
Glucose (mmol/L) after delivery	0.650	0.100–1.201	0.021	1.513	1.109–2.063	0.009	1.511	1.103–2.071	0.010
D dimer (mg/L) after delivery	0.533	−0.109–1.175	0.103	1.145	0.811–1.617	0.442	1.458	1.020–2.086	0.039
Pronation (AIMS)	−1.510	−2.476–−0.543	0.002	0.320	0.180–0.569	<0.001	0.217	0.116–0.406	<0.001
Supination (AIMS)	−3.040	−4.090–−1.990	<0.001	0.188	0.093–0.378	<0.001	0.173	0.086–0.348	<0.001

GWG—gestational weight gain; BMI—body mass index; HTA—hypertension; DM—diabetes mellitus; BT—blood type; UC—urine culture; GBS—group B streptococcus; PROM—premature rupture oft membranes; BDP—fetal biparietal diameter; HC—head circumference; AC—abdominal circumference; FL—femoral length; EFW—estimated fetal weight; AFI—amniotic fluid index; IUGR—intrauterine growth restriction; HGB—hemoglobin; AIMS—Alberta Infant Motor Scale.

**Table 4:** Perinatal predictors for increased prepregnancy BMI and excessive GWG.

Parameters	Multiple Linear Regression Analysis Increased Prepregnancy BMI			Multivariate Logistic Regression Analysis Excessive GWG			Multivariate Logistic Regression Analysis Increased Prepregnancy BMI and Excessive GWG		
	B	95% CI	*p*	Exp(B)	95% CI	*p*	Exp(B)	95% CI	*p*
Age (years)	−0.025	−0.070–0.019	0.264	-	-	-	-	-	-
Weight at delivery (kg)	0.069	0.030–0.108	0.001	-	-	-	-	-	-
Prepregnancy BMI (kg/m^2^)	-	-	-	0.571	0.345–0.945	0.029	-	-	-
GWG (kg)	−1.007	−1.64–0.377	0.002	-	-	-	-	-	-
BMI (kg/m^2^) at delivery	0.725	0.611–0.840	<0.001	2.535	1.664–3.862	<0.001	-	-	-
Parity	-	-	-	-	-	-	-	-	-
Obesity degree	-	-	-	2.862	0.327–25.058	0.342	-	-	-
Allergies	-	-	-	-	-	-	-	-	-
Family anamnesis	0.280	−0.282–0.843	0.326	0.895	0.246–3.254	0.867	0.724	0.225–2.333	0.589
HTA	−0.184	−1.083–0.715	0.687	0.456	0.051–4.043	0.481	7.349	1.396–38.691	0.019
DM	−0.413	−1.042–0.217	0.198	0.791	0.168–3.725	0.767	2.962	0.976–8.987	0.055
Thrombophilia	-	-	-	-	-	-	2.704	0.504–14.505	0.246
Anemia	−0.557	−1.073–−0.040	0.035	1.068	0.244–4.680	0.930	1.824	0.645–5.161	0.257
Drug use in pregnancy	0.352	−0.122–0.827	0.145	1.752	0.504–6.095	0.378	0.901	0.334–2.431	0.837
Menarche	0.067	−0.113–0.247	0.462	-	-	-	-	-	-
BT	-	-	-	-	-	-	-	-	-
UC	-	-	-	-	-	-	-	-	-
GBS	-	-	-	-	-	-	-	-	-
Drug use at delivery	-	-	-	-	-	-	-	-	-
Delivery mode	-	-	-	-	-	-	-	-	-
PROM	-	-	-	-	-	-	-	-	-
Complications at delivery	-	-	-	1.475	0.236–9.231	0.678	-	-	-
Gestational week at delivery	-	-	-	-	-	-	-	-	-
BPD fetal (mm)	-	-	-	1.124	0.869–1.454	0.373	-	-	-
HC fetal (mm)	-	-	-	-	-	-	-	-	-
AC fetal (mm)	0.009	−0.011–0.028	0.384	1.003	0.962–1.046	0.892	1.002	0.980–1.024	0.882
FL fetal (mm)	-	-	-	-	-	-	-	-	-
EFW (grams)	0.000	−0.001–0.001	0.382	1.000	0.997–1.002	0.864	-	-	-
AFI (mm)	0.002	−0.004–0.008	0.560	-	-	-	1.013	1.000–1.027	0.047
IUGR	-	-	-	-	-	-	-	-	-
Newborn length (cm)	-	-	-	-	-	-	-	-	-
Newborn weight (grams)	-	-	-	-	-	-	-	-	-
APGAR 1 min	-	-	-	1.096	0.122–9.853	0.935	-	-	-
APGAR 5 min	-	-	-	0.580	0.030–11.340	0.719	-	-	-
Leucocytes (10^9^/L) before delivery	-	-	-	-	-	-	-	-	-
HGB (gr/L) before delivery	-	-	-	0.952	0.868–1.043	0.287	-	-	-
Thrombocytes (10^9^/L) before delivery	-	-	-	-	-	-	-	-	-
Glucose (mmol/L) before delivery	-	-	-	-	-	-	-	-	-
D dimer (mg/L) before delivery	-	-	-	-	-	-	-	-	-
Leucocytes (10^9^/L) after delivery	-	-	-	-	-	-	-	-	-
HGB (gr/L) after delivery	0.016	−0.006–0.037	0.156	1.055	0.984–1.132	0.133	0.990	0.950–1.032	0.636
Thrombocytes (10^9^/L) after delivery	-	-	-	-	-	-	-	-	-
Glucose (mmol/L) after delivery	0.285	0.031–0.538	0.028	1.516	0.763–3.011	0.235	0.992	0.607–1.622	0.975
D dimer (mg/L) after delivery	-	-	-	-	-	-	1.446	0.928–2.255	0.103
Pronation (AIMS)	−0.099	−0.527–0.328	0.646	0.448	0.155–1.294	0.138	0.397	0.174–0.905	0.028
Supination (AIMS)	−0.258	−0.788–0.272	0.338	0.670	0.180–2.495	0.551	0.369	0.151–0.904	0.029

GWG—gestational weight gain; BMI—body mass index; HTA—hypertension; DM—diabetes mellitus; BT—blood type; UC—urine culture; GBS—group B streptococcus; PROM—premature rupture of membranes; BDP—fetal biparietal diameter; HC—head circumference; AC—abdominal circumference; FL—femoral length; EFW—estimated fetal weight; AFI—amniotic fluid index; IUGR—intrauterine growth restriction; HGB—hemoglobin; AIMS—Alberta Infant Motor Scale.
